# A mutant Tat protein inhibits infection of human cells by strains from diverse HIV-1 subtypes

**DOI:** 10.1186/s12985-017-0705-9

**Published:** 2017-03-14

**Authors:** Lina Rustanti, Hongping Jin, Mary Lor, Min Hsuan Lin, Daniel J. Rawle, David Harrich

**Affiliations:** 1 School of Medicine, the University of Queensland, Herston, QLD 4029 Australia; 2 Department of Cell and Molecular Biology, QIMR Berghofer Medical Research Institute, Herston, QLD 4029 Australia; 3 National Institute of Health Research and Development, the Ministry of Health of Republic of Indonesia, Jalan Percetakan Negara 29, Central Jakarta, 10560 Indonesia; 4 Department of Microbiology and Immunology, Graduate Institute of Biomedical Sciences, College of Medicine, Chang Gung University, Kwei-Shan, Taoyuan, 33302 Taiwan; 5 School of Chemistry and Molecular Biosciences, the University of Queensland, St. Lucia, QLD 4072 Australia

## Abstract

**Background:**

Nullbasic is a mutant HIV-1 Tat protein that inhibits HIV-1 replication via three independent mechanisms that disrupts 1) reverse transcription of the viral RNA genome into a DNA copy, 2) HIV-1 Rev protein function required for viral mRNA transport from the nucleus to the cytoplasm and 3) HIV-1 mRNA transcription by RNA Polymerase II. The Nullbasic protein is derived from the subtype B strain HIV-1_BH10_ and has only been tested against other HIV-1 subtype B strains. However, subtype B strains only account for ~10% of HIV-1 infections globally and HIV-1 Tat sequences vary between subtypes especially for subtype C, which is responsible for ~50% HIV-1 infection worldwide. These differences could influence the ability of Tat to interact with RNA and cellular proteins and thus could affect the antiviral activity of Nullbasic. Therefore, Nullbasic was tested against representative HIV-1 strains from subtypes C, D and A/D recombinant to determine if it can inhibit their replication.

**Methods:**

Nullbasic was delivered to human cells using a self-inactivating (SIN) γ-retroviral system. We evaluated Nullbasic-mCherry (NB-mCh) fusion protein activity against the HIV-1 strains in TZM-bl cell lines for inhibition of transactivation and virus replication. We also examined antiviral activity of Nullbasic-ZsGreen1 (NB-ZSG1) fusion protein against the same strains in primary CD4^+^ T cells. The Nullbasic expression was monitored by western blot and flow cytometry. The effects of Nullbasic on primary CD4^+^ T cells cytotoxicity, proliferation and apoptosis were also examined.

**Results:**

The results show that Nullbasic inhibits Tat-mediated transactivation and virus replication of all the HIV-1 strains tested in TZM-bl cells. Importantly, Nullbasic inhibits replication of the HIV-1 strains in primary CD4^+^ T cells without affecting cell proliferation, cytotoxicity or level of apoptotic cells.

**Conclusion:**

A SIN-based γ-retroviral vector used to express Nullbasic fusion proteins improved protein expression particularly in primary CD4+ T cells. Nullbasic has antiviral activity against all strains from the subtypes tested although small differences in viral inhibition were observed. Further improvement of in γ-retroviral vector stable expression of Nullbasic expression may have utility in a future gene therapy approach applicable to genetically diverse HIV-1 strains.

**Electronic supplementary material:**

The online version of this article (doi:10.1186/s12985-017-0705-9) contains supplementary material, which is available to authorized users.

## Background

The HIV-1/AIDS pandemic remains a huge social and economic burden. By 2014, 36.9 million people were living with HIV and 1.2 million AIDS related death cases were reported [1]. One of the major obstacles in treating this disease is a high genetic diversity of HIV-1 that leads to different rates of disease progression and resistance to antiviral drugs [2, 3]. We have investigated an anti-HIV-1 agent that targets three different steps of virus replication by targeting viral and cellular proteins, and therefore may have efficacy against HIV-1 with diverse genetic backgrounds.

The agent is a Tat mutant protein derived from HIV-1 subtype B strain BH10 that strongly inhibits HIV-1 replication in human cells [4], and is referred to as Nullbasic. Wild type Tat is an essential HIV-1 protein required for transactivation of the HIV-1 long terminal repeat (LTR) promoter resulting in high levels of viral mRNA transcription by RNA polymerase II [5]. It also plays a role in HIV-1 reverse transcription [6, 7] and in other cellular processes such as immune suppression, induction of inflammatory cytokines and apoptosis [8–10]. Nullbasic, which has been described previously [4, 11, 12], has a substitution mutation spanning the entire basic domain; amino acids 49 to 57, RKKRRQRRR, are replaced with GGGGGAGGG. Studies show that Nullbasic expressed in cells is located in the nucleus and cytoplasm [13], and inhibits HIV-1 replication by 1) inhibiting HIV-1 transcription by RNA polymerase II through interaction with the positive transcription elongation factor (p-TEFb) and causing epigenetic silencing of the HIV-1 LTR promoter [4, 12, 13], 2) inhibiting Rev-dependent viral mRNA transport from the nucleus by binding to DEAD/H-box helicase 1 (DDX1) [13, 14], and 3) inhibiting reverse transcription by directly interacting with reverse transcriptase (RT) leading to accelerated uncoating kinetics post-infection and defective viral DNA synthesis [15].

HIV-1 sequence diversity is categorized by HIV-1 subtypes that are defined by comparisons of envelope genes. These subtype variations can also be observed as differences in viral proteins, such as Tat, Rev and RT. Amino acid sequence variation in the viral proteins of various HIV-1 subtypes can affect virus replication and virulence [16]. For example, RT from subtype C isolates differs from subtype B by ~7–10%, which can affect drug susceptibility and cause drug resistance [16]. Tat proteins from different subtypes can vary up to 40% without significantly affecting Tat transactivation ability [17], but the effects on many alternative functions of Tat [18] have not been studied in detail.

To date, Nullbasic antiviral activity has only been tested against HIV-1 subtype B strains such as HIV-1_NL43_ [4, 11]. However, subtype B strains only accounts for ~10% of HIV-1 infections globally and HIV-1 Tat sequences vary between subtypes especially for subtype C, which is responsible for ~50% HIV-1 infection worldwide [19, 20]. Subtype C is predominant in sub Saharan Africa, India and South America [21], while subtype D and recombinant A/D are increasing in sub-Saharan Africa [22, 23]. Whether sequence variations in different HIV-1 subtypes alter the susceptibility to the antiviral effect of Nullbasic has not been examined. Therefore, in this study, Nullbasic ability to inhibit replication of HIV-1 strains from different subtypes including C, D and A/D was evaluated. To enable protein expression detection in the targeted cells, Nullbasic was tested in the form of fusion proteins as NB-mCh [13] or NB-ZSG1 [11].

## Methods

### Cell lines and cultures

HEK 293T (ATCC), TZM-bl [24, 25] and Phoenix-Ampho [26] cell lines were grown in Dubelcco’s modified Eagle’s medium (DMEM; Life Technologies) supplemented with 10% (^v^/_v_) fetal bovine serum (FBS), penicillin (100 IU/ml) and streptomycin (100 μg/ml) (referred to as DF10 medium). TZM-bl expressing NB-mCh or mCh cell lines were established by transduction of NB-mCh or mCh virus-like particles (VLPs) and then selected by fluorescent activated cell sorter (FACS) for the top 10% of mCherry positive cells by mean fluorescent intensity (MFI).

Peripheral blood mononuclear cells (PBMCs) were isolated from healthy donor’s buffy coat supplied by Australian Red Cross Blood service using Ficoll density gradient centrifugation. CD4^+^ cells were isolated from the PBMCs by using a magnetic-activated cell sorting human CD4^+^ cell isolation kit (Miltenyi Biotec) as per the manufacturer’s instruction. The selected cells were grown in 6 cm tissue culture dishes and stimulated using plates pre-coated with purified anti-human CD3 (clone HIT3a) and anti-human CD28 (clone CD28.2) antibodies (BioLegend) in RPMI medium supplemented with 20% (^v^/_v_) FBS and 5 ng/ml interleukin-2 (IL-2) (hereafter called RF20 IL-2) for 2 days. All cells were grown at 37 °C in humidified incubators with 5% CO_2_.

### Plasmids constructs

pSRS11-SF-γC-EGFP was a gift from Axel Schambach and Christopher Baum [27]. pSRS11-SF-γC-NB-mCh or pSRS11-SF-γC-mCh or pSRS11-SF-γC-NB-ZSG1 or pSRS11- SF-γC-ZSG1 construct was made by replacing the enhanced green fluorescent protein gene in pSRS11-SF-γC-EGFP with NB-mCh or mCh or NB-ZSG1 or ZSG1. A proviral plasmid pGCH making HIV-1_NL43_ (GenBank accession number AF324493) was previously described [13]. The proviral plasmid pZAC (GenBank accession number JN188292.1) was obtained from Jochen Bodem [28]. The proviral plasmids pELI and pMAL (Los Alamos accession number A07108 and A07116 respectively) were provided by Damian Purcell [29]. The exon *tat* genes with hemagglutinin epitope were synthesized by GenScript and ligated into pcDNA3.1^+^ plasmid (Thermofisher Scientific).

### HIV-1 and VLPs production

HIV-1 subtype B, C, D and A/D were produced from pGCH, pZAC, pELI and pMAL proviral plasmids respectively. HEK 293 T cells were grown on a 10 cm plate at ~80% confluency and transfected with 10 μg of each proviral plasmid then incubated for 24 h at 37 °C. On the next day, the transfected cells were washed with 1 x phosphate buffered saline (PBS) and the DF10 media was replaced. The supernatant containing HIV-1 VLPs was collected 48 and 72 h post transfection and the amount of HIV-1 capsid (CA) protein in each supernatant was measured by enzyme-linked immunosorbent assay (ELISA) (Zeptometrix) as recommended by the manufacturer.

NB-mCh or mCh or NB-ZSG1 or ZSG1 VLPs were produced in Phoenix-amphotropic retroviral packaging producer cell line by co-transfection of 7.5 μg of pSRS11-SF-γC vector expressing NB-mCh or mCh or NB-ZSG1 or ZSG1 and 1.5 μg of Gag-Pol expressing plasmid using X-tremeGENE^TM^ DNA transfection reagent (Roche) in a 10 cm plate. Six hours post transfection, the cells were washed with PBS and the media was replaced. The VLPs were collected 48 and 72 h post transfection and filtered through a 0.45 μm filter.

### Western blot analysis

Cell lysates were made from 5 × 10^6^ NB-mCh or mCh-TZM-bl cells, or from 3 × 10^6^ CD4-NB-ZSG1, CD4-ZSG1 or non-transduced CD4 cells in cell lysis buffer (50 mM Tris HCl pH 7.4, 150 mM NaCl, 1 mM ethylenediaminetetraacetic acid and 1% (^v^/_v_) Triton X-100). The total protein concentration was measured by a Bradford assay using Bio Rad protein assay (Bio Rad) and equivalent amounts of protein were used for analysis. The blots were stained with a anti-mCherry rabbit antibody (BioVision), a rabbit anti-Tat antibody (Diatheva), a mouse anti-ZsGreen1 (Origene), a rabbit anti-β-tubulin antibody (Sigma Aldrich), or a goat anti-actin antibody (Santa Cruz) as indicated. Appropriate species-specific secondary antibodies conjugated to horse radish peroxidase (HRP) (Cell Signaling Technology) and followed detection by chemiluminescence (BioRad).

### Transactivation assay

Tissue culture dishes (6 cm) were seeded with 5 × 10^5^ TZM-bl cells expressing NB-mCh or mCh and then co-transfected with 1 μg of each subtype Tat plasmid or pCDNA3.1^+^ without an insert and 150 ng of Gaussia luciferase expression plasmid. After 48 h, the cells were washed with PBS and cell lysates were made using Glo Lysis buffer (Promega). Luciferase assays were performed in 96 well white polystyrene microplates as per the manufacturer’s instructions using 10 μl of the cell lysates and Dual-Glo® luciferase substrate (Promega). Luciferase activity in each sample was measured within 20 min by using a luminescence microplate reader and relative values were normalized to Gaussia luminescence in the sample.

Next, 3 × 10^5^ TZM-bl cells expressing NB-mCh or mCh or non-transduced (NT) TZM-bl cells were seeded in 6 well plates. The next day, the cells were infected with HIV-1_NL4–3_ (subtype B), HIV-1_ZAC_ (subtype C) [28], HIV-1_ELI_ (subtype D) and HIV-1_MAL_ (A/D recombinant subtype) virus supernatant containing 20 ng of CA, or a mock supernatant for 48 h. The cells were washed with PBS and then cell lysates were made using Glo Lysis buffer (Promega). Luciferase activity was measured as described above.

### Transduction of NB-ZSG1 or ZSG1 VLP in CD4^+^ T cells

NB-ZSG1 or ZSG1 VLPs were concentrated using the precipitation method through the addition of 20% (^v^/_v_) of 34% polyethylene glycol 8000 (Sigma Aldrich) and 10% (^v^/_v_) of 0.3 M NaCl solution. The solution mixture was incubated at 4 °C for 1.5 h, mixed every 30 min and then centrifuged at 1500 × g for 1 h at 10 °C. The supernatant was discarded and the precipitate was resuspended in 600 μl RF20 IL-2 medium. The concentrated VLP (150 μl) was added to Retronectin (Takara) coated 24 well plate and incubated at 37 °C for 30 min. 5 × 10^5^ stimulated CD4+ cells were added to each well and incubated for 3 days. Transduced cells were processed by FACS to collect ZSG1 positive cells which were grown for 3 days further. The RF20-IL2 media was replaced every day.

### Infection of HIV-1_NL4–3_ (subtype B), HIV-1_ZAC_ (subtype C), HIV-1_ELI_ (subtype D) and HIV-1_MAL_ (A/D recombinant subtype) in TZM-bl cell lines and primary CD4^+^ T cells

TZM-bl cells expressing NB-mCh or mCh or NT (3 × 10^5^ cells/well) cultured in a 6 well plate were infected with a virus stock containing 20 ng CA of HIV-1_NL4–3_, HIV-1_ELI_ and HIV-1_MAL_ or 40 ng CA of HIV-1_ZAC_, or a mock supernatant for 2 h at 37 °C. A larger amount of HIV-1_ZAC_ was required to yield measurable infections. The virus was then removed by washing the cells 3 times with PBS and the infected cells were cultured at 37 °C with 5% of CO_2_. The culture supernatants were sampled on day 3 and 5 post infection. The amount of HIV CA present was measured using a CA ELISA kit (Zeptometrix) according to the manufacturer’s instruction.

Primary CD4^+^ T cells (5 × 10^5^ NB-ZSG1 or ZSG1 or NT) were infected with virus stocks containing 2 ng CA of each HIV-1 subtype for 2 h at 37 °C. After infection, the cells were washed with PBS and cultured in RF20 IL-2 medium. Cell and supernatant samples were collected on days 0, 3, 7, 10 and 14 by centrifugation at 500 × g for 5 min. The amount of viral CA in the supernatant was measured by ELISA. The cells were fixed with 1% paraformaldehyde in PBS solution and NB-ZSG1 or ZSG1 expression was measured by flow cytometry.

### Cytotoxicity assays

Cell metabolic activity was measured by MTS assay using a CellTiter 96® aqueous one solution cell proliferation reagent (Promega) according to the manufacturer’s instructions. Cell proliferation was quantified using a Violet Proliferation Dye 450 (BD Horizon^TM^) assay in accordance with the manufacturer’s instructions and violet fluorescence was measured using a violet laser-equipped BD LSRFortessa^TM^ IV flow cytometer. Apoptosis events were quantified using a PE Annexin V apoptosis detection kits (BD Pharmingen^TM^) as per the manufacturer’s instructions. Camptothecin, which induces apoptosis in CD4+ T cells, was used as a positive control.

### Statistical analysis

Mean values of percentage of transactivation inhibition between strains were compared using Kruskal-Wallis one-way analysis of variance. A 95% confidence interval was used, therefore a *p* value less than 0.05 was considered to be significant.

## Results

### Inhibition of transactivation and replication of HIV-1 strains from diverse subtypes in TZM-bl cells by NB-mCh fusion protein

**Fig. 1:**
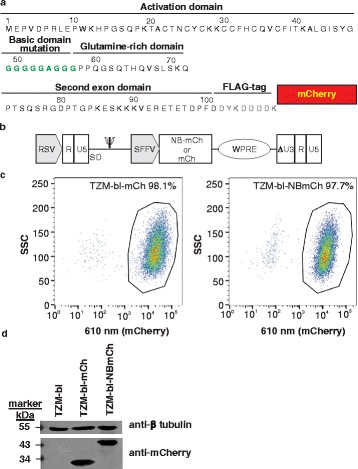
Expression of Nullbasic-mCherry (NB-mCh) and mCherry (mCh) in TZM-bl cells. **a** A schematic of the NB-mCh amino acid sequence. The basic domain of wild type Tat was mutated to glycine or alanine residues as shown (*green*) and other domains are labeled. **b** A schematic diagram of SIN γ-retroviral SRS11-SF-γC vector showing NB-mCh or mCh that are expressed by a SFFV internal promoter. Rous Sarcoma Virus (RSV) promoter is used to transcribe mRNA for packaging in VLPs. The woodchuck hepatitis virus post transcription regulatory element (WPRE) was inserted adjacent to NB-mCh or mCh to enhance gene expression. **c** Purity of selected TZM-bl cells expressing NB-mCh or mCh analyzed by flow cytometry. **d** NB-mCh and mCh expression in TZM-bl cells was detected by a Western Blot using anti-mCherry antibody. The blot was also stained with an anti-β tubulin antibody

A schematic diagram of Nullbasic protein and the basic domain mutations (amino acids 49–57) to glycine and an alanine are shown in Fig. 1a. A NB-mCh fusion protein, used previously to investigate Nullbasic inhibition on HIV-1 Rev activity [14], was inserted into a SIN γ-retroviral vector pSRS11-SF-γC (Fig. 1b) [27]. VLPs produced in Phoenix amphotropic packaging cells [30] were used to transduce TZM-bl cells and those stably expressing NB-mCh or mCh (hereafter referred to as TZM-bl-NB-mCh or TZM-bl-mCh, respectively) were collected by FACS. Expression of NB-mCh and mCh was confirmed by flow cytometry analysis of the purified cells (Fig. 1c) and western blot analysis using an anti-mCherry antibody (Fig. 1d).

**Fig. 2:**
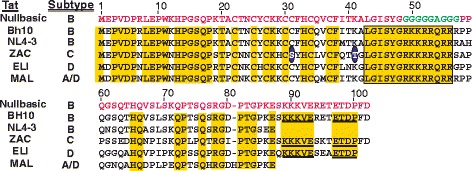
A comparison of Nullbasic amino acid sequence against an amino acid sequence alignment for Tat_BH10_, Tat_NL4–3_, Tat_ZAC_, Tat_ELI_ and Tat_MAL_. Amino acids highlighted in yellow are conserved for the Tat variants. Tat_ZAC_ amino acid residue S31 and T41 are circled (*blue*)

It is worth reviewing the amino acid sequence heterogeneity of Tat from these different strains by comparing the alignment of Tat proteins from HIV-1_NL4–3_ (subtype B), HIV-1_ZAC_ (subtype C) [28], HIV-1_ELI_ (subtype D) and HIV-1_MAL_ (A/D recombinant subtype). The four different amino acid sequences have amino acid residue substitutions in all domains except for amino acids 43 to 56 (Fig. 2, boxed), which includes the basic domain, completely conserved. The first 20 residues of each Tat protein include 17 positions that are conserved or functionally similar. The carboxyl terminal amino acids 90 to 100 of subtype C and D Tat proteins have conserved amino acid sequences KKKVE and ETDP (Fig. 2, underlined), which are also present in Nullbasic (derived from HIV-1_BH10_). All four Tat proteins maintained the cysteine residues in the cysteine-rich domain with one exception; HIV-1_ZAC_ (subtype C) has a C31S substitution.

Tat mediates HIV-1 transactivation by binding to trans-activation response (TAR) RNA in the R region of HIV-1 LTR and recruiting P-TEFb [31] that then binds a super elongation complex (SEC) [32, 33]. P-TEFb consists of cyclin T1 and cyclin dependent kinase 9 (CDK9) [31]. In Tat, K41 is important for intramolecular hydrogen bonding and structural integrity of the Tat core [34] and is present in all Tat proteins shown except HIV-1_ZAC_ which has T41. A crystal structure of the Tat-P-TEFb complex showed that the surface of 37% amino acids 1–49 are complementary to the kinase complex, and this model indicates that the interactions between Tat and P-TEFb can accommodate substitutions commonly present in different Tat genes [34]. However, Tat interacts with other cellular proteins many of which are important for HIV transcription [35]. Therefore, it is possible that these subtle differences could affect the ability of Nullbasic to inhibit the transactivation by Tat from the HIV-1 strains shown.

**Fig. 3:**
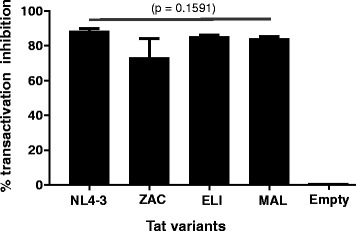
NB-mCh inhibits Tat-mediated transactivation after transfection of TZM-bl by Tat from different strains. TZM-bl cell lines expressing NB-mCh or mCh were transfected with pCDNA3.1+ containing each Tat variant or with empty pCDNA3.1^+^ A Gaussia luciferase expression plasmid was co-transfected as a control for transfections efficiency. Luciferase activity was measured 48 h post transfection. Bars indicate mean percentage of transactivation inhibition by NB-mCh compared to mCh cells from two independent experiments each carried out in triplicate. The *p* value of the data set is shown

To test if transcription by Tat from different HIV-1 strains can be inhibited by Nullbasic, TZM-bl-NB-mCh and TZM-bl-mCh cell lines stably carrying a HIV-1-LTR firefly luciferase reporter were co-transfected with eukaryotic expression plasmids that express either Tat_NL4–3_, Tat_ZAC_, Tat_ELI_, Tat_MAL_ or an empty expression plasmid and a Gaussia luciferase expression plasmid to control for transfection efficiency. Tat can activate the HIV-1-LTR firefly luciferase reporter in TZM-bl cell lines, while Gaussia luciferase reporter enables linear quantification of relative transfection efficiencies between samples [36]. The amount of firefly luciferase activity present in lysates from the transfected TZM-bl cells was measured and the relative luminescence unit (RLU) values were normalized to Gaussia luciferase RLUs measured in the culture supernatant. The results show that expression of NB-mCh in TZM-bl-NB-mCh cells reduced transactivation of the HIV-1 LTR luciferase reporter in all subtypes tested from ~70 to ~90% (Fig. 3). Although Tat_ZAC_ showed a trend towards less inhibition than others, this difference did not achieve statistical significance. Transfection of an empty expression plasmid did not affect the level of luciferase made by TZM-bl-NB-mCh cells compared to TZM-bl-mCh cells. Overall, the level of Nullbasic inhibition of the Tat proteins tested here was similar to a previous report [4].

**Fig. 4:**
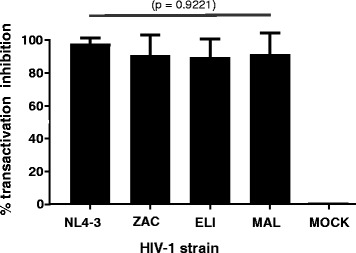
NB-mCh inhibits Tat-mediated transactivation after infection of TZM-bl by different HIV-1 subtypes. TZM-bl cell lines expressing NB-mCh or mCh were infected with each HIV-1 strain or with mock supernatant. Luciferase activity was measured 48 h post infection and normalized to total protein in the sample. *Bars* indicate mean percentage of transactivation inhibition by NB-mCh compared to mCh cells. *Error bars* indicate standard deviation of three independent experiments carried out in triplicate. The *p* value of the data set is shown

Post infection, the integrated provirus produced Tat can activate the HIV-1-LTR luciferase reporter in TZM-bl cells, which is an indicator of virus infection and replication. To evaluate Nullbasic inhibition of Tat-mediated transactivation of different HIV-1 strains, we infected TZM-bl-NB-mCh and TZM-bl-mCh cells with HIV-1_NL4–3_ (subtype B), HIV-1_ZAC_ (subtype C), HIV-1_ELI_ (subtype D) and HIV-1_MAL_ (recombinant A/D subtype) produced in HEK293T cells or with mock supernatant. Lysates prepared from HIV-1 infected TZM-bl cells 48 h post infection were assayed for firefly luciferase activity and the results were normalized to total protein concentration in the cell lysates. NB-mCh significantly reduced the amount of RLUs produced compared to control lysates made from infected TZM-bl-mCh cells. HIV-1_NL4–3_ viral Tat transactivation of the LTR-luciferase reporter was inhibited by ~97%; slightly lower inhibitions of transactivation were measured for all other HIV-1 strains; HIV-1_ZAC_ by ~90%, HIV-1_ELI_ by ~89% and HIV-1_MAL_ by ~91% (Fig. 4). However, the difference between transactivation inhibition in HIV-1_NL4–3_ and other strains did not achieve statistical significance. The level of luciferase made by mock-infected TZM-bl-NB-mCh cells compared to TZM-bl-mCh cells was unchanged. The combined experiments demonstrate that NB-mCh can inhibit transactivation of the TZM-bl LTR reporter by each Tat tested albeit at a slightly reduced level compared to HIV-1_NL4–3_.

**Fig. 5:**
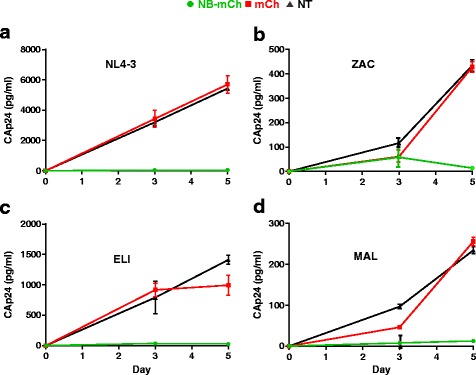
NB-mCh inhibits virus replication of all the HIV-1 strains tested in TZM-bl cells. TZM-bl cell lines expressing NB-mCh or mCh or NT were infected with each HIV-1 strain; **a** HIV-1_NL4–3_ (subtype B), **b** HIV-1_ZAC_ (subtype C), **c** HIV-1_ELI_ (subtype D) and **d**. HIV-1_MAL_ (recombinant A/D subtype). After 3 and 5 days infection, culture supernatant was assayed for HIV-1 CA by ELISA. The experiment was performed in triplicate and mean values and standard deviations are shown from two independent experiments

Finally, we tested if the presence or absence of NB-mCh could inhibit viral replication (rather than assessing effects on the integrated HIV-1 LTR-luciferase reporter in the cell line) of each HIV-1 strain in the TZM-bl cell lines. In this 5 day experiment, detection of CA by ELISA requires virus replication. Therefore, TZM-bl-NBmCh and TZM-bl-mCh cells were infected, supernatants were collected on day 3 and 5 post infection, and the amount of CA in each sample was by measured by ELISA. As shown in Fig. 5a-d, the data shows that each HIV-1 strain replicated in NT TZM-bl and TZM-bl-mCh cells with an increasing level of CA from day 3 to 5. However, HIV-1 replication of all strains was inhibited in TZM-bl-NBmCh cells. Comparing day 5 CA levels by TZM-bl-mCh to TZM-bl-mCh cells showed the production of HIV-1 CA levels dropped by >99% for HIV-1_NL4–3_, ~97% for HIV-1_ZAC_, ~98% for HIV-1_ELI_, and ~97% for HIV-1_MAL_. As previously observed, the trend was that NB-mCH inhibited HIV-1_NL4–3_ better than other strains tested. Nevertheless, the combined results support the hypothesis that Nullbasic can inhibit virus replication of the HIV-1 strains tested in TZM-bl cells.

### Antiviral activity of Nullbasic-ZsGreen1 (NB-ZSG1) in primary CD4^+^ T cells against 4 HIV-1 subtypes

We previously used an MLV-based γ-retroviral vector, pGCsamEN [11], containing NB-ZSG1 or ZSG1 to transduce primary CD4^+^ cells. In those experiments, cells that expressed NB-ZSG1 significantly delayed HIV-1_NL4–3_ replication compared to cells expressing ZSG1. However, pGCsamEN is not a SIN vector and expression of a transgene is via the MLV-LTR promoter. Non-SIN γ-retroviral vector can be transcriptionally repressed in cells [37], which can be lessened by SIN-based γ-retroviral vectors that used strong constitutively expressed internal promoters. Therefore, SRS11-SF-γC-NB-ZSG1 VLPs were used to transduce CD4^+^ T cells in this study. The transduced CD4^+^ T lymphocytes were selected by FACS using parameters previously described and a western blot was performed to confirm NB-ZSG1 and ZSG1 expressions (hereafter referred to as CD4-NB-ZSG1 and CD4-ZSG1, respectively) in the sorted cells (see Additional file 1).

**Fig. 6:**
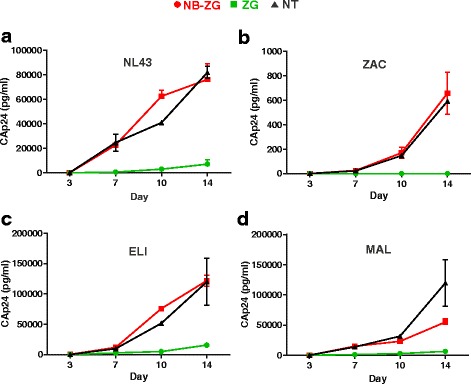
NB-ZSG1 inhibits virus replication of all the HIV-1 strains tested in primary CD4^+^ T cells. CD4^+^ T cells (NT) or CD4^+^ T cells expressing NB-ZSG1 or ZSG1 were infected with each HIV-1 strain; **a** HIV-1_NL4–3_ (subtype B), **b** HIV-1_ZAC_ (subtype C), **c** HIV-1_ELI_ (subtype D) and d. HIV-1_MAL_ (recombinant A/D subtype). The experiment was performed in triplicate and culture supernatant from each replicate was assayed by ELISA for CA on day 3, 7, 10 and 14 post infection. Mean values and standard deviations from two independent experiments at each time point are shown

CD4-NB-ZSG1, CD4-ZSG1 and NT CD4 cells were infected with each HIV-1 strain and virus replication was monitored for 14 days (Fig. 6). All HIV-1 strains were inhibited but some strain specific differences were noted. For example, HIV-1_NL4–3_, HIV-1_ZAC_, and HIV-1_ELI_ were strongly inhibited. At day 14 post infection, in CD4-ZSG1 to CD4-NB-ZSG1 cells, CA levels reduced by 90% for HIV-1_NL4–3_, and 88% for HIV-1_ELI_ and HIV-1_MAL_. However, no CA was detected after infection with HIV-1_ZAC_. It is worth noting that based on CA expression levels, this HIV-1_ZAC_ replicated poorly in TZM-bl (Fig. 5b) and CD4^+^ T cells (Fig. 6b). Compared to HIV-1_NL43,_ HIV-1_ZAC_ replication in CD4+ T cells on day 14 post infection was ~100-fold lower. A reduced level of HIV-1_ZAC_ replication compared to HIV-1_NL4–3_ was reported previously [28]. Therefore, this may account for a lack of detectable CA by HIV-1_ZAC_ in CD4-NB-ZSG1 cells.

**Fig. 7:**
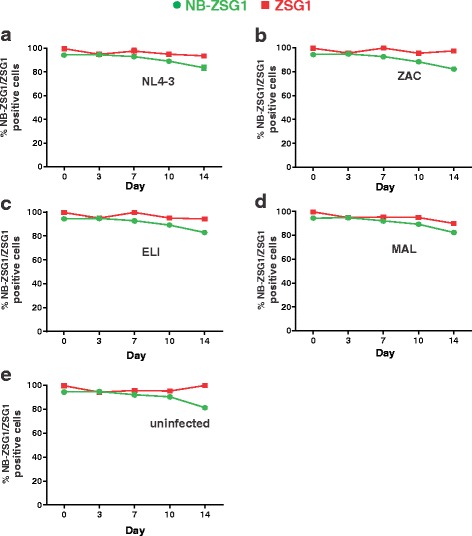
A temporal analysis of NB-ZSG1 or ZSG1 expressed in CD4^+^ T cells. The percentage of NB-ZSG1 or ZSG1 positive cells in the CD4^+^ T cells population infected by **a** HIV-1_NL4–3_ (subtype B), **b** HIV-1_ZAC_ (Subtype C), **c** HIV-1_ELI_ (Subtype D) **d** HIV-1_MAL_ (A/D recombinant subtype) and **e** uninfected CD4^+^ T cells was monitored by flow cytometry on day 3, 7, 10 and 14 post infection. Mean values and standard deviations from two independent experiments performed in triplicates are shown

NB-ZSG1 and ZSG1 expression was monitored in uninfected and HIV-1-infected CD4^+^ T cells by flow cytometry. CD4^+^ T cell populations were initially collected by FACS so that ZSG1 positive cell population was >97%. The percentage of ZSG1 positive cells in the population by 14 days ranged from 90% (for HIV-1_ELI_) to 99% (uninfected CD4^+^ T cells) in all experiments, whereas NB-ZSG1 levels were approximately 82% in uninfected and infected CD4+ T cells (Fig. 7). However, NB-ZSG1 expression reported here was much higher than in previous experiments using pGCsamEN vectors where the percentage of CD4^+^ T cells expressing NB-ZSG1 ranged from 40 to 50% of cells [11].

**Fig. 8:**
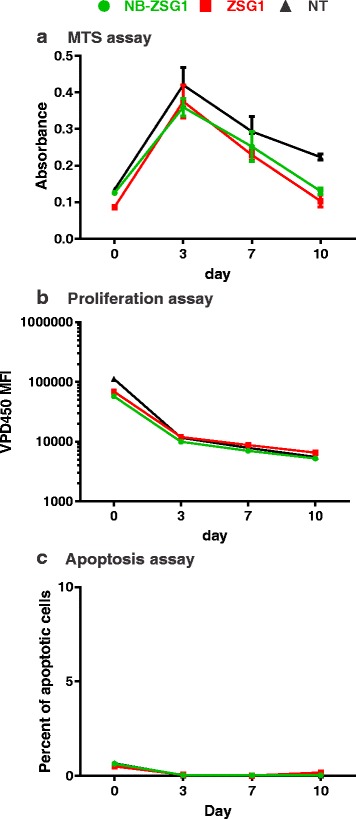
NB-ZSG or ZSG expression does not affect CD4^+^ T cell viability, proliferation or induce apoptosis. **a** An MTS assay was used to measure the viability of CD4^+^ T cells expressing NB-ZSG1 or ZSG1 or NT. **b** The proliferation of CD4^+^ T cells expressing NB-ZSG1 or ZSG1 or NT was measured using VPD450 dye assay. **c** An apoptosis assay using PE Annexin V was used to monitor levels of apoptosis in CD4^+^ T cells expressing NB-ZSG1 or ZSG1 or NT. All assays were performed in triplicate and mean values and standard deviations are shown

We investigated if high level expression of NB-ZSG1 in CD4^+^ T cells had a measurable detrimental effect that could explain why levels of NB-ZSG1 positive cells declined. First, cell viability was evaluated by an MTS colorimetric assay. This assay measures cellular metabolism based on reduction of the MTS tetrazolium compound by NAD(P)H-dependent oxydoreductase enzymes largely in the cytosolic compartment of dividing cells [38, 39]. No significant difference between CD4-NBZSG1 and CD4-ZSG1 cells were found, although both NB-ZSG1 and ZSG1 cells had a slightly lower metabolic activity than NT cells, suggesting the transduction, cell purification processes or ZSG1 were responsible (Fig. 8a). Second, we measured cell proliferation using a cell permeable VPD450 dye for monitoring of cell division by flow cytometry. No difference in proliferation was observed between CD4-NB-ZSG1 and CD4-ZSG1 (Fig. 8b). Given that HIV-1 wild type Tat is reported to have pro- and anti-apoptotic activities in primary CD4^+^ T cells (reviewed in [40]), an annexin V assay was used to determine if NB-ZSG1 expression affected cellular apoptosis. All of the cells tested had very little or no apoptosis (Fig. 8c), therefore we cannot account for a drop in NB-ZSG1 levels in CD4^+^ T cells due detrimental effects of NB-ZSG1 on the cellular pathways tested. However, Nullbasic may affect other cellular pathways other than those tested leading to reduced levels of Nullbasic expression in the cells.

In summary, here we show that Nullbasic can inhibit replication of HIV-1 strains from different subtypes. The outcome indicates that the replication pathways affected by Nullbasic are most likely shared by these HIV-1 strains.

## Discussions

In this study, we investigated if Nullbasic could inhibit viral gene expression and virus replication of four different strains representing four HIV-1 subtypes in human cells. We previously showed that Nullbasic has three independent antiviral properties at different stage of HIV-1 replication cycle [4]; 1) inhibition of transactivation of virus gene expression by HIV-1 Tat [4, 11], 2) inhibition of HIV-1 Rev activity by sequestration of DDX1 [12, 13], and 3) binding to HIV-1 reverse transcriptase in the virion leading to premature uncoating and defective reverse transcription in newly infected cells [14]. Given that Nullbasic inhibits HIV-1 by binding to both cellular (P-TEFb and DDX1) and viral (RT) targets, we found that, as expected, NB fusion proteins had antiviral activity against all strains tested although some small differences were observed.

Using TZM-bl cells, the effect of Nullbasic on transactivation and virus replication was examined in three ways. Briefly, wild type Tat mediates HIV-1 transactivation by binding and recruiting the SEC and P-TEFb (composed of cyclin T1 and CDK9) to nascent viral mRNA where CDK9 can phosphorylate RNA polymerase II leading to highly processive RNA transcription [31]. A crystal structure of the Tat-P-TEFb complex showed that Tat tightly binds to P-TEFb as 37% of its folded N-terminal domain (amino acids 1–49) surface is complementary to the kinase. In Nullbasic, amino acids 1–48 are wild type but amino acids 49–57 are mutated. Hence, Nullbasic is able to bind P-TEFb [13], but not recruit the protein complex to nascent viral mRNA, which requires the RNA binding function of the Tat basic domain (amino acids 49–57) [8].

Firstly, in TZM-bl transfection experiments where equivalent amounts of each Tat expression plasmid were used, the overall inhibition of transactivation had a similar range (70–90% inhibition), but Tat_ZAC_ was consistently inhibited the least by NB-mCh. Interestingly, a consensus subtype C Tat was reported to have superior transactivation capacity compared to a consensus subtype B Tat [41], whereas Tat_ZAC_ was a weaker transcriptional activator here compared to the other Tat proteins tested. This may be due to a Tat_ZAC_ K41T substitution that may affect Tat_ZAC_ structure and interaction between Tat_ZAC_ and P-TEFb [8]. Secondly, TZM-bl cells were infected by each HIV-1 strain and transactivation of the TZM-bl LTR-luciferase reporter was inhibited at similar levels (~90% inhibition of Tat_ZAC_, Tat_ELI_ and Tat_MAL_). Thirdly, replication of all four viral strains was strongly inhibited by NB-mCh, which ranged from 97 to 99%. It is interesting that NB-mCh inhibited virus replication of each strain better than it inhibited transactivation LTR-Luciferase reporter, but the reason for this is unclear. It could be due to Nullbasic effects on Rev activity or reverse transcription, or perhaps differences in the TAR RNAs of the various viral strains. Further work will be required to elucidate the reasons. The data clearly shows that viral transcription and replication mediated by the four different Tat variant proteins, representing different HIV-1 subtypes, was inhibited by NB-mCh under the conditions tested.

The replication of all HIV-1 strains in stimulated primary CD4^+^ T cells was also inhibited by Nullbasic, but differences were noted here too. The replication of HIV-1_ZAC_ in the presence of NB-ZSG1 was below the limit of detection (~4 pg/ml), whereas HIV-1_NL4–3_, HIV-1_ELI_ and HIV-1_MAL_ were strongly inhibited. We also noted that although the FACS isolated CD4-NB-ZSG1 and CD4-ZSG1 cells were >97% ZSG1 positive, the percentage of ZSG1 positive cells was maintained by CD4-ZSG1 population over 14 days whereas the percentage of ZSG1 positive cells in the CD4-NB-ZSG1 population decreased by about 10–20%. Our data indicates that cell proliferation of CD4-NB-ZSG1 and CD4-ZSG1 are similar, and levels of cytotoxic effects and apoptotic cells were unchanged as well. NB-ZSG1 may affect cellular pathways other than those assayed. For example, we recently reported that NB-ZSG1 strongly suppressed HIV-1 transcription in Jurkat cells [12], so one possible cause of this NB-ZSG1 decreased expression level is that NB-ZSG1 also negatively affects transcription by the constitutively active spleen focus-forming virus (SFFV) promoter. Given that NB-ZSG1 is able to target P-TEFb, it may impede an SEC required for HIV-1 transcription [42], and perhaps SEC complexes that stimulate transcription by the SFFV promoter as well. Testing these possibilities will require determining if the NB-ZSG disrupts the P-TEFb-SEC complexes, and further understanding of transcriptional activation of the SFFV promoter.

Our data shows that HIV-1 replication increased in CD4-NB-ZSG1 cells as the percentage of NB-ZSG1 positive CD4 T cells decreased, as we observed previously [11]. It is possible that alternative promoters used to express Nullbasic in the retroviral vector may provide sustained expression of NB-ZSG1, and lead to better viral control. In addition, it would be also interesting to introduce a Nullbasic-type mutation into other Tat variants and test if a strain-specific custom Nullbasic gene is a better inhibitor of specific strains.

## Conclusions

SIN-based γ-retroviral vectors improved expression of Nullbasic and inhibited HIV-1 replication. The study shows that Nullbasic can inhibit replication of the HIV-1 strains from different HIV-1 subtypes tested in TZM-bl cells line as well as in primary CD4^+^ T cells. Stable expression of Nullbasic may have utility in a future gene therapy approach applicable to genetically diverse HIV-1 strains.

## Additional file

Additional file 1: Expression of NB-ZSG1 and ZSG1 in CD4^+^ T cell lysates detected by Western Blot. Cell lysates prepared from non-transduced CD4^+^ T cell (NT), CD4-NB-ZSG1 and CD4-ZSG1 cells were assayed by SDS-PAGE and Western Blot. The blots were probed with anti-Tat, anti-ZSG1 and anti-β-tubulin antibodies as indicated, which were detected using appropriate HRP-conjugated secondary and chemiluminescence. (PDF 24 kb)

## Abbreviations

CA: Capsid
DDX1: DEAD/H-box helicase 1
ELISA: Enzyme-linked immunosorbent assay
FACS: Fluorescent activated cell sorter
FBS: Fetal bovine serum
HRP: Horse radish peroxidase
IL-2: Interleukin-2
LTR: Long terminal repeat
MFI: Mean fluorescent intensity
NB-mCh: Nullbasic-mCherry
NB-ZSG1: Nullbasic-ZsGreen1
NT: Non-transduced
PBMCs: Peripheral blood mononuclear cells
PBS: Phosphate buffered saline
p-TEFb: Positive transcription elongation factor
RLU: Relative luminescence unit
RT: Reverse transcriptase
SEC: Super elongation complex
SFFV: Spleen focus-forming virus
SIN: Self-inactivating
TAR: Trans-activation response
VLP: Virus-like particle
WPRE: Woodchuck hepatitis virus post transcription regulatory element
