# Supplementary material for: A mutant Tat protein inhibits infection of human cells by strains from diverse HIV-1 subtypes
Source: Virol J. 2017 Mar 14;14:52. doi: 10.1186/s12985-017-0705-9 (PMC5348743; doi:10.1186/s12985-017-0705-9)
Supplement: Additional file 1: — Expression of NB-ZSG1 and ZSG1 in CD4+ T cell lysates detected by Western Blot. Cell lysates prepared from non-transduced CD4+ T cell (NT), CD4-NB-ZSG1 and CD4-ZSG1 cells were assayed by SDS-PAGE and Western Blot. The blots were probed with anti-Tat, anti-ZSG1 and anti-β-tubulin antibodies as indicated, which were detected using appropriate HRP-conjugated secondary and chemiluminescence. (PDF 24 kb) [file 12985_2017_705_MOESM1_ESM.pdf]

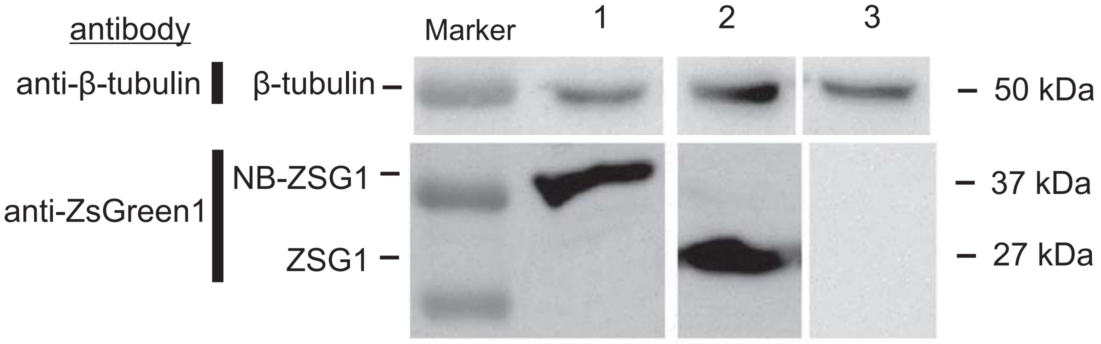

Additional file 1. Western blot of cell lysates prepared from CD4+ T cells expressing; 1. NB-ZSG1, 2. ZSG1 or 3. non-transduced cells using anti-β-tubulin or anti-ZsGreen1 antibodies.
